# High-throughput approaches for precision medicine in high-grade serous ovarian cancer

**DOI:** 10.1186/s13045-020-00971-6

**Published:** 2020-10-09

**Authors:** Meinusha Govindarajan, Christoph Wohlmuth, Matthew Waas, Marcus Q. Bernardini, Thomas Kislinger

**Affiliations:** 1 Department of Medical Biophysics, University of Toronto, Toronto, Canada; 2 Division of Gynecologic Oncology, Princess Margaret Cancer Centre, University Health Network, Toronto, Canada; 3 Department of Obstetrics and Gynecology, Paracelsus Medical University, Salzburg, Austria; 4 Princess Margaret Cancer Centre, University Health Network, Toronto, Canada; 5 Department of Obstetrics and Gynaecology, University of Toronto, Toronto, Canada

**Keywords:** High-grade serous carcinoma, High-throughput technology, Liquid biopsies, Therapeutic targets, Molecular subtypes

## Abstract

High-grade serous carcinoma (HGSC) is the most prevalent and aggressive subtype of ovarian cancer. The large degree of clinical heterogeneity within HGSC has justified deviations from the traditional one-size-fits-all clinical management approach. However, the majority of HGSC patients still relapse with chemo-resistant cancer and eventually succumb to their disease, evidence that further work is needed to improve patient outcomes. Advancements in high-throughput technologies have enabled novel insights into biological complexity, offering a large potential for informing precision medicine efforts. Here, we review the current landscape of clinical management for HGSC and highlight applications of high-throughput biological approaches for molecular subtyping and the discovery of putative blood-based biomarkers and novel therapeutic targets. Additionally, we present recent improvements in model systems and discuss how their intersection with high-throughput platforms and technological advancements is positioned to accelerate the realization of precision medicine in HGSC.

## Background

The American Cancer Society estimates that in 2020, 21,750 women will be newly diagnosed with ovarian cancer in the USA and ~ 13,940 will die from the disease [1]. Epithelial ovarian cancer (EOC) represents the fifth most common cause of cancer death overall and is the leading cause of death from gynecologic malignancies in the USA, Canada and Europe [1–3]. EOC is a heterogeneous disease with different types of histologies, molecular and microenvironmental features [4]. Histologically, EOC is traditionally classified into five major subtypes: high-grade serous (HGSC), low-grade serous (LGSC), clear cell, endometrioid and mucinous ovarian cancer [4]. A more recent classification model categorizes EOC into type I and II tumors, where LGSC, endometrioid, mucinous and clear cell carcinomas are classified as type I [5–7]. These neoplasms typically present as large, unilateral, cystic tumors and clinically tend to behave in an indolent fashion [7]. Genetically, type I cancers are characterized by minor chromosomal instability and may harbor *BRAF*, *KRAS* and *PTEN* mutations. Type II cancers, on the other hand, comprise of HGSC, which account for the vast majority of all EOCs, carcinosarcomas and undifferentiated carcinomas. HGSCs have a high degree of genetic instability and are characterized by the presence of acquired or inherited mutations in different DNA repair pathways including *TP53, BRCA1/2* and other defects in homologous recombination repair genes [4, 5, 7]. Recent evidence suggests that HGSC originates from the fimbriae of the fallopian tube secondarily involving the ovary and peritoneum [8].

Early-stage disease is typically asymptomatic, and currently there are no proven screening strategies for HGSC that reduce mortality [9, 10]. The tumor volume in the ovaries is substantially less than that of type I tumors, and 80% of HGSCs are diagnosed at advanced disease stages [7, 11]. Even in advanced HGSC, symptoms are nonspecific and include back pain, fatigue, bloating, constipation, abdominal pain, change in bowel function, urinary symptoms and weight loss [12]. The initial diagnostic work-up includes a pelvic ultrasound or computed tomography (CT) and (CA125) assessment [13]. Magnetic resonance imaging may be used to further stratify pelvic masses, and a CT of the thorax, abdomen and pelvis is performed for staging purposes. The standard of care treatment for HGSC is primary debulking surgery (PDS) to no visible residual disease with adjuvant platinum-based chemotherapy. Two randomized trials comparing PDS and chemotherapy with neoadjuvant chemotherapy followed by interval debulking surgery showed similar survival, but both studies had minimal residual disease and survival rates in both study arms [13–15]. Despite recent advances, approximately 70% of EOCs recur and the 5-year survival rate for metastatic disease remains poor at 30% [1, 16].

**Fig. 1:**
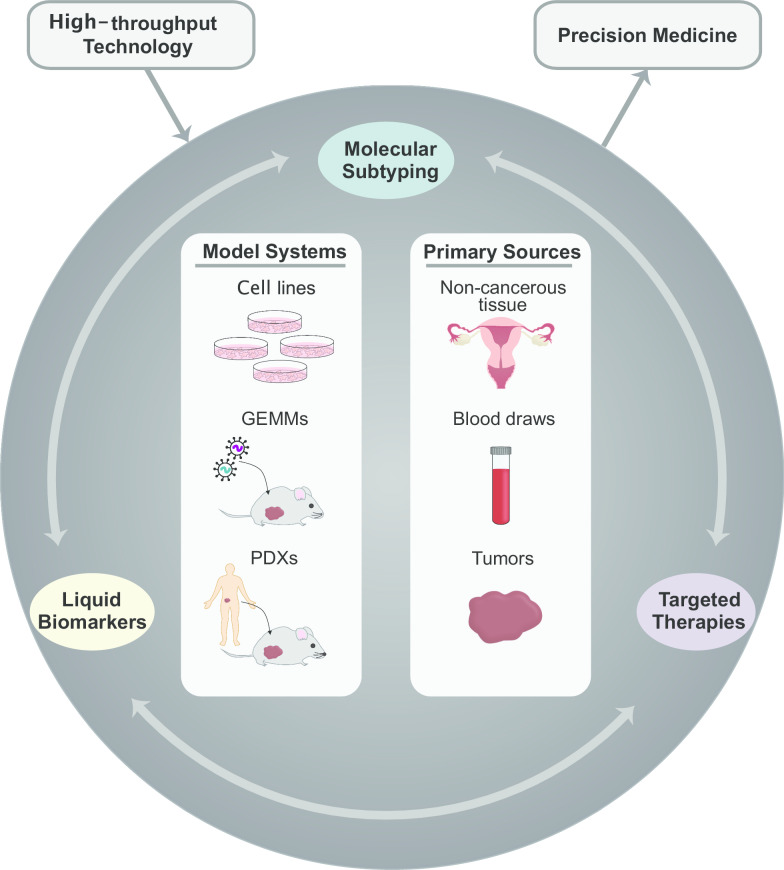
Applications of high-throughput technologies for precision medicine. High-throughput examination of experimental models and patient samples is promising for molecular subtyping and the discovery of liquid biomarkers and targeted therapies, which cumulatively contribute to advancing precision medicine in HGSC. *GEMM* genetically engineered mouse model, *PDX* patient-derived xenograft

Precision medicine refers to the notion of tailoring clinical management of diseases to account for patient heterogeneity. Although it is well known that EOC comprises several pathologically distinct diseases, the current standard of care is to generally manage these subtypes as a single entity. Molecular screening has revealed a vast degree of variability within the HGSC subtype itself [17]. This is reflected in the array of clinical outcomes as not all patients respond to conventional therapies. This underlying complexity also makes it unlikely that a single tumor marker will be effective for all patients. The use of poly (ADP-ribose) polymerase (PARP) inhibitors for patients with *BRCA1/2* mutations is an example of the shift toward precision medicine in HGSC; however, additional work is still necessary. Biomarkers that are reflective of tumor burden and therapies which target specific tumor characteristics are needed to improve patient outcomes. Advancements in high-throughput biological techniques have provided new opportunities for the discovery of biomarkers and therapeutic targets. These platforms allow for the simultaneous profiling of thousands of molecules and the subsequent generation of a wealth of biological data. Together with large cohorts of well-annotated patient samples and improved model systems, these approaches have facilitated novel insights into biological heterogeneity at an unprecedented scale. In this review, we provide an overview of how high-throughput approaches have contributed to the molecular profiling of patient heterogeneity within HGSC and highlight the utility of these technologies in the discovery of putative blood-based biomarkers and therapeutic targets as a step toward enabling precision medicine as a reality for all HGSC patients (Fig. 1). We also discuss the complementary role of HGSC experimental models in advancing these discoveries.

## Molecular tumor profiling of HGSC

High-throughput molecular profiling of tumor samples has been used to gain insights into the biological aberrations underlying the pathogenesis of HGSC. The largest study in mapping the molecular features of HGSC was conducted by The Cancer Genome Atlas (TCGA) network, where 489 tumor samples were subjected to genomic and transcriptomic analyses [17]. Exome sequencing detected *TP53* mutations in 96% of tumors. Interestingly, subsequent histological analysis of the *TP53* wild-type tumors in this cohort revealed differences in morphological features indicating that these tissues were not truly HGSC tumors [18], suggesting the proportion of *TP53* mutations to be even higher than reported. This finding is consistent with other reports of ubiquitous *TP53* mutations in HGSC [19]. Serous tubal intraepithelial carcinomas (STIC) (the precursor lesion of HGSC) and ‘p53-signature lesions’ (the hypothesized precursor of STIC) in the fallopian tube have been shown to share identical *TP53* mutations to HGSC, signifying that *TP53* mutations develop early in the HGSC carcinogenic process [20]. Germline and somatic mutations in *BRCA1* and *BRCA2* are the next most prevalent mutations in HGSC, cumulatively present in 22% of the TCGA cohort [16]. Seven other significantly mutated genes were identified albeit only in 2–6% of cases, demonstrating a limited mutational landscape in HGSC. In contrast, HGSC exhibits a high degree of chromosomal instability evident by extensive copy number alterations (CNAs) in each tumor and the identification of 113 significantly recurrent CNAs throughout the entire cohort [17]. The TCGA study also revealed that half of HGSC tumors had genomic and/or epigenetic deficiencies in homologous recombination, further underscoring the role of erroneous DNA repair mechanisms in HGSC pathogenesis [17]. Indeed, homologous repair deficiency (HRD) is a crucial determinant of platinum sensitivity in HGSC [21]. Other frequently altered pathways in HGSC include *RB1*, *PI3K/RAS*, *NOTCH* and *FOXM1* [17]. In an attempt to deconvolute this vast genomic heterogeneity, Macintyre et al. [22] have recently identified seven copy number signatures in HGSC, some of which were found to be associated with previously mentioned mutations, aberrant pathways and survival outcomes, yet larger studies are still required to validate these associations.

The profiling of mRNA expression in HGSC tumors has identified four overlapping transcriptional subtypes of HGSC: C1—mesenchmyal, C2—immunoreactive, C4—differentiated and C5—proliferative [17, 23]. Independent studies have identified prognostic implications associated with these subtypes in which the immunoreactive subtype exhibited improved survival outcomes, whereas the mesenchymal and proliferative subtypes demonstrated the worst overall survivals [24, 25]. Building on these consistent findings, Leong et al. [26] have identified a gene signature consisting of 39 differentially expressed genes for classification of these subtypes. The Clinical Proteomic Tumor Analysis Consortium (CPTAC) analyzed the global proteomes of 169 HGSC tumors from the TCGA cohort [27]. Clustering of tumors based on protein abundance revealed five subtypes, four of which demonstrated a clear resemblance to the classical transcriptomic subtypes and one novel subtype classified as stromal [27]. Integration of proteomic and CNA data revealed that proteins associated with multiple CNAs were enriched in cell invasion/migration and immune processes, suggesting there is a functional convergence of the high degree of chromosomal instability [27]. The low overall correlation between mRNA expression and protein expression in this investigation highlights the importance of multi-omic profiling to achieve a comprehensive understanding of molecular alterations underlying HGSC [27].

Aside from delineating molecular heterogeneity between patients, high-throughput tumor profiling can also be used to elucidate the diversity within a tumor. Deconvolution of bulk HGSC transcriptional data has revealed that individual tumors often display multiple subtype signatures [25], accentuating the additional layer of molecular complexity offered by intratumor heterogeneity. Albeit on a small scale, recent efforts in multiregion tumor profiling of HGSC tumors have uncovered intratumor molecular heterogeneity in both a spatial manner and temporal manner [26, 28–31]. Although larger investigations are warranted to extend the generalizability of these data, these studies highlight the susceptibility of bulk subtypes to sampling bias and the potential confounding role of stromal components in tumor profiling. Additionally, in a disease characterized by extensive intraperitoneal dissemination, multiregion molecular profiling of primary tumors and metastases can be of value for discerning the biology underlying HGSC progression [32–34]. Despite the loss of spatial microenvironment context, single-cell technologies can also provide insights into intratumor heterogeneity [35, 36]. Further large-scale studies using these emerging approaches may shed light into how intratumor heterogeneity manifests in clinical outcomes such as therapeutic resistance. Overall, the spectrum of molecular differences within HGSC underscores the significance in using high-throughput approaches to further understand the biological abnormalities and translate these findings into novel biomarkers and targeted therapies.

## Blood-based biomarkers for HGSC

A biomarker is a measurable feature that is reflective of biological processes and can provide information regarding the disease state of an individual. Cancer biomarkers are used for various purposes throughout the course of disease progression, including assessing the likelihood of developing cancer, diagnosing malignancies, determining prognosis, predicting patient responses to specific therapies and monitoring residual disease post-treatment and during remission. In contrast to directly examining tumor tissue, liquid biopsies can facilitate minimally invasive tumor assessments to guide clinical decisions. Blood is an attractive biological fluid for biomarkers in clinical practice due to the standardized collection procedures and abundant availability. In this section, we briefly review the current landscape of HGSC blood-based biomarkers and discuss the utility of high-throughput approaches in the discovery of novel biomarkers to help improve clinical management of HGSC.

## Liquid biopsies in clinical practice

In the context of HGSC, and EOC in general, serum biomarkers are currently used for the differential diagnosis of a pelvic mass prior to surgery, monitoring response to treatment and detecting recurrent disease. Although definitive diagnosis of EOC currently requires histological examination, differential diagnosis of a pelvic mass determines preoperative referral [37]. This is crucial as optimal tumor resection and subsequent improved outcomes are more likely when surgical management for EOC is performed by gynecological oncologists rather than general surgeons or gynecologists [38]. In addition to determining treatment efficacy and prognosis following therapy, accurate markers of treatment response are also used to evaluate novel therapies in clinical trials [39]. Considering the high rates of HGSC relapse, early detection of recurrent disease is imperative for appropriate timing of therapies to improve survival [40].

### Cancer antigen 125

CA125 is a large membrane glycoprotein encoded by the gene *MUC16* and was identified as a tumor marker for EOC in 1983 [41]. Significant expression of CA125 is observed in 85% of serous, 65% of endometrioid, 40% of clear cell, 36% of undifferentiated and 12% of mucinous ovarian cancers, highlighting the lack of utility of CA125 in some EOC subtypes [42]. Despite being the most widely used biomarker for EOC, CA125 offers limited value as a diagnostic test. Serum concentrations of CA125 are elevated in 90% of advanced-stage EOCs and less than 50% of early-stage EOCs, resulting in a low sensitivity for detecting early-stage disease [43]. Furthermore, serum CA125 abundance has a low specificity for EOC as levels can be increased due to multiple benign gynecological and medical conditions including endometriosis and pregnancy [44]. The low specificity is especially manifest in premenopausal women who are at an increased risk of many of these other conditions [45]. Given the limitations of CA125 as a stand-alone diagnostic marker, the Risk of Malignancy Index (RMI) [46] and the International Ovarian Tumor Analysis (IOTA) Adnex model [47] were developed to integrate serum CA125 levels, ultrasound criteria and demographics, resulting in improved specificity and sensitivity for differential diagnosis of pelvic masses prior to surgery. When evaluated as a potential screening test, both the UKCTOCS study [9] and the PLCO trial [48] demonstrated that serum testing of CA125 alone or combined with transvaginal ultrasound imaging did not reduce mortality due to EOC and resulted in an increase in unnecessary invasive procedures associated with complications, underlining the clinical consequences of low specificity.

Nevertheless, CA125 offers clinical utility when evaluating treatment response and monitoring remission. A decrease in serum CA125 is indicative of treatment response, whereas a persistence of abnormally elevated CA125 or increases may suggest treatment resistance and/or residual disease [39]. Many post-treatment surveillance protocols include serial measurements of CA125, as rising serum CA125 is strongly predictive of disease recurrence [49–51]. A rise in CA125 concentration has been shown to precede clinical detection of recurrent disease by at least three to five months [49, 52, 53]. However, up to half of patients within the normal limits of CA125 during remission are found to have small volumes of disease during a second-look surgery [54, 55]. Hence, despite being the earliest sign of recurrence currently available, CA125 is not optimally sensitive for detecting recurrence in all patients.

### Human epididymis protein 4

Human epididymis protein 4 (HE4), encoded by the gene *WFDC2*, is a secreted glycoprotein that is overexpressed in serous and endometrioid ovarian cancers [56]. Hellstrom et al. [57] initially determined that serum HE4 was comparable to CA125 for distinguishing between patients with advanced-stage disease and healthy controls. Subsequent studies have produced conflicting reports regarding the sensitivity of HE4 compared to CA125 as a diagnostic test, yet there is a consensus that HE4 is more specific than CA125, especially in premenopausal women [58–61]. This superiority is likely due to serum levels of HE4 being less influenced by other gynecological disorders such as endometriosis [62]. Serum HE4 is also elevated in at least a third of patients who do not demonstrate elevated serum CA125 levels, signifying a role for complementary markers in diagnostics [63]. Serum HE4 is currently approved to be used as a tumor marker for monitoring disease progression and recurrence. A study evaluating serum levels of HE4 and CA125 prior to surgery for suspicious recurrent EOC found HE4 to be more sensitive and specific than CA125 [64]. In a pilot study, Anastasi et al. [65] found that a rise in serum HE4 preceded elevated serum CA125 five to eight months in five out of eight patients with recurrent disease. Preliminary studies have also revealed that HE4 elevation can detect recurrence in a subset of EOC patients that do not present with increased serum CA125 [66, 67]. The combination of both markers resulted in a higher sensitivity and specificity in detecting recurrence than either marker alone [68], but data from larger prospective trials, including potential benefits in survival, are still pending.

### Multimarker assays

In response to the promising data regarding the complementary potential between CA125 and HE4 as diagnostic markers, the Risk of Malignancy Algorithm (ROMA) was developed and is currently approved for differential diagnosis [69]. ROMA combines serum measurements of CA125 and HE4 and uses two different logarithmic regression models based on menopausal status to determine the likelihood of malignancy in women who are having surgery for pelvic masses [70]. A meta-analysis comparing ROMA, HE4 and CA125 revealed that ROMA demonstrated the greatest sensitivity and HE4 exhibited the highest specificity for differential diagnosis, although these differences were not statistically significant [71]. In a direct comparison, ROMA was found to be more sensitive than RMI with similar specificities [72], yet both assays demonstrated low sensitivity for early-stage disease [73]. OVA1 is a biomarker panel of five proteins used for the differential diagnosis of pelvic masses prior to surgery. The test consists of immunoassays for two upregulated proteins (CA125, beta 2 microglobulin) and three down-regulated proteins (transferrin, transthyretin, apolipoprotein A1) in serum [74]. An algorithm is used to integrate the measurements of each marker to generate an ovarian malignancy risk score ranging from 0 – 10. The threshold for risk of malignancy is dependent on menopausal status. Many prospective studies comparing the performance of OVA1 to CA125 have reported higher sensitivity than CA125, especially for early-stage disease, yet lower specificity [75–77]. There have been no direct comparisons between OVA1 and ROMA to date. Overa is a second-generation multivariate assay which was intended to overcome the low specificity of OVA1 [78]. Overa uses serum measurements of CA125, HE4, apolipoprotein A1, transferrin and follicle-stimulating hormone to assess the likelihood of malignancy in women who will undergo surgery for a pelvic mass. The incorporation of follicle-stimulating hormone eliminates the need for assessing menopausal status as in OVA1. Overa was designed and validated using the same study population as OVA1, thus allowing for direct comparisons between the two assays. Indeed, Overa demonstrated an improved specificity and similar sensitivity to OVA1 [78].

### Germline BRCA1/2 mutations

Germline deficiencies in *BRCA1/2* are the strongest genetic risk factors for nonmucinous EOC [79]. The cumulative risk of developing ovarian cancer in *BRCA1* and *BRCA2* carriers ranges from 40 to 59% and 16 to 18%, respectively [80–82]. As such, genetic counseling and genetic testing can be suggested for patients with familial history of breast, ovarian, pancreatic or prostate cancer to identify those who are at an elevated risk [83] and will likely benefit from preventative measures such as risk-reducing bilateral salpingo-oophorectomies [84, 85]. In addition, germline and somatic *BRCA1/2* mutations are considered predictive biomarkers due to strong associations with favorable outcomes following both platinum-based chemotherapy [86, 87] and maintenance therapy with PARP inhibitors [88, 89]. PARP inhibitors have also been approved for usage as monotherapy in *BRCA1/2*-deficient women with recurrent disease [90, 91]. Hence, genetic testing for *BRCA1/2* mutations is recommended for all newly diagnosed ovarian cancer patients to aid in therapy selection and determining cancer risk for family [83].

## Emerging high-throughput biomarker discovery approaches

**Fig. 2:**
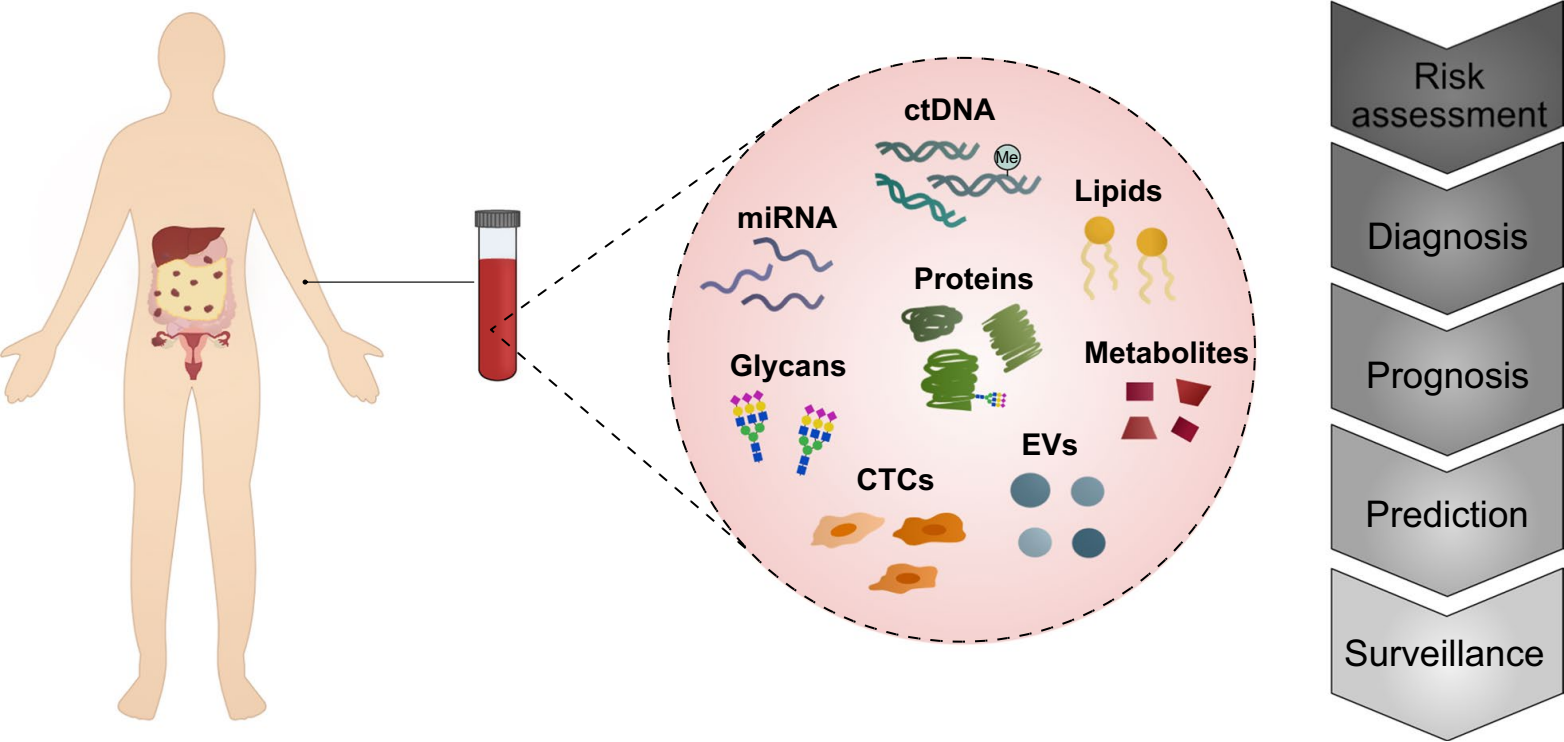
Utility of liquid biopsies. High-throughput platforms have enabled the profiling of several classes of circulating molecules as putative noninvasive tumor markers. These molecules can be informative for various purposes throughout the clinical course of cancer progression. *CTC* circulating tumor cell, *ctDNA* circulating tumor DNA, *EV* extracellular vesicle, *miRNA* microRNA

Though blood is an attractive source of biomarkers, limitations in the throughput of highly sensitive molecular measurements have been a challenge, especially for heterogeneous diseases such as HSGC. Advancements in ‘omics’ approaches have enabled the ability to characterize and evaluate various classes of circulating molecules as potential blood-based biomarkers (Fig. 2). A biomarker discovery pipeline is typically initiated with a discovery phase in which large-scale comparative profiling experiments of blood, tumor tissue or model systems are used to generate a list of candidate markers. Following the discovery phase, targeted methods of quantification are often applied to validate candidate markers in clinical samples [92]. Here, we discuss the various classes of molecules and types of discovery approaches which have been applied to HSGC biomarker discovery.

### Circulating tumor DNA

Circulating cell-free DNA (cfDNA) are short fragments of DNA released into the bloodstream from apoptotic or necrotic cells [93]. The quantification of total cfDNA has revealed that EOC patients have elevated levels of cfDNA compared to healthy controls and patients with benign disease [94–96]. However, evaluation of cfDNA abundance is not a direct measure of tumor burden as DNA fragments are also released by noncancerous cells. The fraction of cfDNA that originated from tumor cells, termed circulating tumor DNA (ctDNA), can be distinguished by the presence of cancer-specific alterations [97]. Given the low abundance of ctDNA compared to cfDNA, highly sensitive approaches such as digital polymerase chain reaction (PCR) and targeted next-generation sequencing (NGS) are used to detect cancer-specific modifications. Since *TP53* mutations are ubiquitous in HGSC, detection of *TP53* mutants in cfDNA has been preferentially used when investigating ctDNA as a biomarker [98–101]. Parkinson et al. [98] analyzed *TP53* mutations in longitudinal plasma samples from HGSC patients undergoing treatment to evaluate the value of ctDNA in determining prognosis and response to treatment. This study revealed that the abundance of *TP53* mutant ctDNA fractions prior to treatment significantly correlated with volumetric measurements of tumors from CT images, unlike CA125, and that a decrease of > 60% of *TP53* mutant ctDNA fractions following treatment was a predictor of time-to-progression. Christie et al. [102] recently investigated whether reversal of germline *BRCA1/2* mutations can be detected in ctDNA, as this molecular alteration is known to correspond with acquired chemo-resistance. Reversion mutations were detected in the plasma of three out of five patients with reversion mutations observed in tumor samples, all of whom were resistant to platinum-based therapy or PARP inhibitors. The authors noted that detection of reversal mutations was associated with the fraction of ctDNA out of total cfDNA, measured by the presence of *TP53* mutant alleles. Certainly, a limiting factor of utilizing ctDNA as a tumor marker is that current detection strategies may not be sensitive enough to detect rare mutants in early-stage disease where the ctDNA fraction is low [103].

In addition to investigating specific genes, examination of genome-wide chromosomal aberrations in ctDNA can be promising for the discovery of novel tumor markers. Harris et al. [104] used whole-genome sequencing to characterize genomic rearrangements in primary tumors of HGSC patients and investigated whether patient-specific aberrant chromosomal junctions could be detected in plasma. ctDNA with patient-specific chromosomal alterations was detected in pre-surgically drawn plasma samples for eight out of ten patients. Postsurgical detection of ctDNA was specific to the only three patients with clinically documented residual disease, suggesting a potential for personalized markers of tumor burden [104]. As changes in DNA methylation and chromatin remodeling play a role in tumor biology, the rise of epigenetic technologies (e.g., methylation profiling) is also promising for the use of ctDNA as blood-based tumor markers [105]. In the context of HGSC, Widschwednter et al. [106] identified aberrant methylation signatures in tumor tissues and developed a three-marker DNA methylation panel for ctDNA that was able to discriminate patients from healthy women or women with benign masses. The panel was also shown to better distinguish between platinum responders and nonresponders than CA125.

### microRNAs

microRNAs (miRNAs) are a class of short (19–25 nucleotides) noncoding RNAs that are involved in gene regulation. miRNAs can function as oncogenes or tumor suppressors depending on cellular context, and expression has been shown to be deregulated in several cancers [107]. miRNAs are actively secreted from cells by binding to protein complexes or by being packaged into extracellular vesicles, thus providing protection from RNAse digestion and degradation in various extreme conditions (e.g., high temperatures, severe pH and multiple freeze–thaw cycles) [108, 109]. The stability of miRNAs in blood renders them as attractive molecules for tumor markers in liquid biopsies. In large-scale miRNA biomarker discovery experiments, high-throughput qRT-PCR panels, microarrays and more recently, NGS, can be used for profiling miRNAs in patient samples [110–112]. Todeschini et al. [110] used microarrays to profile miRNA expression in sera from HGSC patients and healthy controls. The differentially expressed miRNAs were then quantified in an independent cohort from which a single miRNA that demonstrated the greatest ability in discriminating HGSC patients from controls was identified as a putative diagnostic biomarker. Shah et al. [111] used qRT-PCR panels for serum miRNA profiling and demonstrated that combining measurements of circulating miRNAs and CA125 can be predictive of surgical resection outcomes for women with HGSC, suggesting value for circulating miRNAs as prognostic markers.

### Proteins

Proteins are the primary functional elements of most biological processes, and thus, protein expression is often deregulated in disease states. Mass spectrometry (MS) is a powerful approach for protein measurement as current MS-based proteomic experiments can detect thousands of proteins in a single sample. MS has already proven to be fruitful in EOC biomarker discovery as the four markers in OVA1 (excluding CA125) were discovered using MS-based approaches [74]. In the studies that led to the development of OVA1, seven protein candidates were identified in the original discovery phase, yet verification of candidates was limited to only those proteins which had existing immunoassays [113, 114]. Though this approach is advantageous for faster clinical adoption, antibody availability can pose as a bottleneck for validation of candidate markers in biomarker discovery pipelines. Targeted MS approaches such as multiple reaction monitoring (MRM) and the more recently developed parallel reaction monitoring (PRM) can enable high-throughput robust quantification independent of antibody availability, thus circumventing the need for antibody development during biomarker discovery.

Detection of blood-based protein markers is challenging due to the large dynamic range and high sample complexity of serum/plasma. Considering that the 22 most abundant proteins in plasma account for 99% of the total mass of protein [115], detection of low-abundance proteins, often the most promising proteins for biomarker candidates, is hindered. Several preanalytical workflows have been developed to overcome this complexity, including the depletion of high-abundance proteins, sample fractionation and/or the enrichment for sub-proteomes [116]. *N*-glycosylation is a posttranslational modification that plays an important role in the stability, solubility and localization of proteins to the cell surface [117]. *N*-glycosylated proteins can be enriched from biological samples using chemoproteomic- and lectin-based approaches [118, 119]. As *N*-glycosylation is highly prevalent among extracellular proteins (including secreted proteins) and is not present on several high-abundance blood proteins (i.e., albumin), the *N*-glycoproteome represents a clinically relevant sub-proteome for liquid biomarker discovery. Sinha et al. [120] recently devised an integrated *N*-glycoproteomics-based approach for detecting biomarkers of HGSC relapse. *N*-glycosylated peptides were enriched from the sera and tumors from recurrent HGSC patient-derived xenograft (PDX) mice and from sera of non-engrafted mice. Species mapping was used to distinguish between peptides of human (tumor) and mouse (stroma) origin, and comparative analysis was used to select a set of candidate markers. Subsequently, PRM was used to quantify the candidates in longitudinal HGSC patient serum samples, revealing four candidates that demonstrated an earlier rise between the remission and the recurrence time points than CA125. Although large-scale clinical validation of the markers is warranted, this study is a proof-of-concept for the use of *N*-glycoproteomics and PDX models in serum protein biomarker discovery for HGSC.

### Glycans, lipids and metabolites

Posttranslational modifications and metabolic processes are important determinants of cellular signaling and modulating phenotypes. As such, these molecular classes also represent promising candidates for blood-based biomarker discovery. Considering that aberrant glycosylation occurs during malignant transformation [121], one such approach consists of profiling differences in glycan structures on glycoproteins. Biskup et al. [122] used MS to compare the serum *N*-glycome profiles between serous EOC patients and healthy women. This glycomics study revealed a marker panel comprising 11 differentially abundant glycans that demonstrated an improved specificity for distinguishing patients from healthy controls compared to CA125. Moreover, as metabolic alterations have been implicated in tumorigenesis [123], metabolomics and lipidomics have emerged as potential avenues for biomarker discovery. Zhou et al. [124] used MS to examine the metabolite profiles of sera from HGSC patients, women with benign ovarian masses and healthy controls and subsequently developed a machine-learning algorithm for diagnostic classification based on the mass spectrum profiles. Buas et al. [125] performed lipidomics analyses of plasma collected from serous EOC patients and patients with benign ovarian masses. A classification model incorporating CA125 and four lipid metabolites demonstrated an increased diagnostic accuracy compared to CA125 alone. Together, these studies suggest a potential utility for plasma metabolites to aid in the diagnosis of HGSC.

### Extracellular vesicles and circulating tumor cells

Aside from investigating freely circulating molecules in blood, molecular profiling of extracellular vesicles (EVs) and circulating tumor cells (CTCs) are alternative approaches for blood-based biomarker discovery. EVs, such as exosomes, are secreted from most cell types, play a role in intercellular communication and contain molecular content from the cell-of-origin [126]. In a pilot study, Taylor et al. [127] identified eight exosomal miRNAs that demonstrated significantly distinct expression profiles in the sera of serous EOC patients compared to the sera of women with benign disease. These exosomal miRNAs exhibited a similar expression profile in tumor tissue and were not detected in the sera of healthy controls. Recently, Kobayashi et al. [128] used microarrays to profile miRNAs in exosomes isolated from the conditioned media of HGSC cell lines and immortalized ovarian surface epithelial cells. A single upregulated miRNA was selected for subsequent quantification in EOC patient sera and was found to be differentially expressed between sera of HGSC patients and sera from non-HGSC patients [128]. This illustrates a potential for noninvasive molecular stratification of EOC. Peng et al. [129] compared the proteomes of serum exosomes from serous EOC patients and tumor tissue, revealing 35 proteins commonly upregulated in comparison with normal samples. These findings suggest that exosomes may be of use for noninvasive molecular tumor examinations.

CTCs are tumor cells that are shed into vasculature and play an important role in metastasis [130]. Although CTCs have been explored as noninvasive tumor markers in the general context of EOC, to the best of our knowledge, there are no published investigations specifically focusing on HGSC to date. Studies have primarily focused on the detection and/or the enumeration of CTCs as potential biomarkers in EOC, yet there have been conflicting reports potentially due to differences in isolation strategies [131–133]. Molecular investigations of CTCs are less prevalent and have traditionally involved the use of qRT-PCR to evaluate the expression of a few specific genes. Zhang et al. [134] examined the expression of six genes that were known to be associated with EOC and demonstrated that *EpCAM* and *ERBB2* expressions in CTCs were correlated with platinum resistance and overall survival. Emerging technologies in microfluidics-based CTC isolation and single-cell molecular analysis present new avenues for high-throughput examinations of individual CTCs. Single-cell RNA sequencing of CTCs has been shown to be promising in understanding clonal resistance and metastasis to potentially inform therapeutic decisions in other cancers [135, 136]. Furthermore, MS-based workflows have recently been developed to profile the proteomes of CTCs, allowing for another layer of molecular characterization [137, 138]. Although single-cell approaches have yet to be applied to CTCs in HGSC, it is proposed that comprehensive molecular characterization of CTCs can provide noninvasive insights regarding intratumor heterogeneity and aid in patient selection for targeted therapies.

## Targeted therapies for HGSC

Targeted therapies are therapeutic agents that act on specific molecular targets, pathways or aspects of the tumor microenvironment that drive the cancer phenotype, in an effort to reduce harm in normal cells. Contemporary systemic management of EOC has progressed from chemotherapy to combination treatments and frontline targeted therapy, when appropriate. In this section, we review the current application of targeted therapies in HGSC clinical practice and describe high-throughput biological workflows for therapeutic target discovery.

## Current clinical use of targeted therapies

**Fig. 3:**
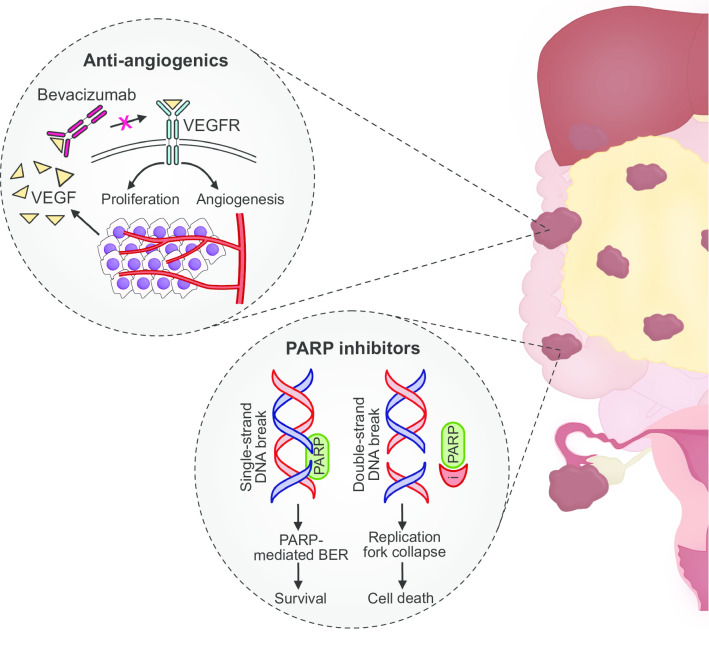
Current targeted therapies for high-grade serous ovarian cancer. **a** Anti-angiogenic agents. Cancer cells secrete vascular endothelial growth factor (VEGF) A that binds to vascular endothelial growth factor receptor (VEGFR) to promote angiogenesis and proliferation. Bevacizumab is a monoclonal antibody which inhibits the binding of VEGF to VEGFR, thus hindering angiogenesis and tumor growth. **b** Poly(ADP-ribose) polymerase (PARP) inhibitors. PARP enzymes mediate base excision repair of DNA single-strand breaks. Inhibition of PARP results in the accumulation of single-strand breaks culminating in DNA double-strand breaks. In cells with homologous repair deficiencies, double-strand breaks are not repaired resulting in replication fork collapse, chromosome instability and cell death. *BER* base excision repair, *PARP* poly (ADP-ribose) polymerase, *VEGF* vascular endothelial growth factor, *VEGFR* vascular endothelial growth factor receptor

Although there are several emerging therapies under clinical investigation for EOC (e.g., immunotherapies [139] and folate receptor-targeted therapies [140]), we have limited our review on the targeted therapies with the most clinical data and they have been approved for use in the clinic (Fig. 3).

### Anti-angiogenic agents

Angiogenesis is a rate-limiting step in the evolution of cancer [141] and has therefore been studied as a potential target for systemic treatment. Bevacizumab is a monoclonal antibody that targets vascular endothelial growth factor (VEGF) A which is secreted by tumors to induce the formation of new blood vessels [142]. Early studies of bevacizumab have demonstrated improved progression-free (PFS) and overall survival (OS) in colorectal and renal cancer [143, 144]. Two landmark trials that assessed the role of concurrent and maintenance treatment with bevacizumab in EOC were the GOG-0218 (primary endpoint: PFS) and the ICON7 (primary endpoints: PFS and OS) studies [145, 146]. Both trials have shown significant improvements in PFS in the intention-to-treat populations with bevacizumab compared to chemotherapy alone but have failed to improve OS in the overall study population. The clinical significance of a three-month difference in PFS has been debated, and as such, bevacizumab is not universally used in the first-line treatment of EOC. In the ICON7 trial, women with high-risk features (inoperable stage III, suboptimal debulking and stage IV disease) randomized to bevacizumab had a significant improvement in mean OS of 4.8 months [147]. Similarly, a subanalysis of the GOG-0218 study suggested that patients with International Federation of Gynecology and Obstetrics (FIGO) stage IV disease may have an increased survival benefit from bevacizumab [148].

In addition to its utility as a first-line therapy, bevacizumab has proven to be effective in patients with recurrent disease. Clinical trials have revealed significant improvements in PFS when bevacizumab was added compared to chemotherapy alone in both platinum-sensitive [149] and platinum-resistant patients [150]. Similar to primary disease, the use of bevacizumab for recurrent disease was not associated with significant improvement in OS for all participants [150, 151]. Other anti-angiogenic therapeutics currently under investigation for EOC include pazopanib [152] and nintedanib [153], both of which have shown similar improvements in progression-free survival in clinical trials. Considering the potential severe side effects including hypertension, renal complications, hemorrhage, gastrointestinal perforation and fistula formation, appropriate patient selection and balancing the risks and potential benefits play a pivotal role for anti-angiogenic treatment.

### Poly (ADP-ribose) polymerase inhibitors

PARP-1 was first described in 1966 [154] but its pivotal role for ovarian cancer was only recently discovered [155]. PARP-1 and PARP-2 are enzymes that play a critical role in base excision repair, a repair mechanism for DNA single-strand breaks [156]. The inhibition of PARP results in an accumulation of single-strand breaks, which can lead to double-strand breaks during replication. The double-strand breaks are normally repaired by a process termed ‘homologous recombination’ [156]. In cancer cells with *BRCA1/2* mutations or other HRDs, the inhibition of PARP results in a synthetic lethal interaction as the accumulation of double-strand breaks coupled with inadequate repair mechanisms can lead to chromosomal instability, cell cycle arrest and subsequent apoptosis [157]. Olaparib, niraparib and rucaparib are the three PARP inhibitors that are currently FDA-approved for recurrent ovarian cancer after showing consistent improvement in PFS [88, 158–160].

In the SOLO-1 trial, olaparib was tested as a frontline maintenance treatment in women with newly diagnosed FIGO stage III–IV ovarian cancer with germline or somatic *BRCA1/2* mutation following cytoreductive surgery and platinum-based chemotherapy. PFS was significantly improved in the olaparib arm compared to placebo and the 3-year progression-free survival was 60.4% versus 26.9%, HR 0.3 (*p* < 0.001) [89]. The FDA has subsequently approved olaparib for frontline maintenance treatment in women with platinum-responsive ovarian cancer and *BRCA1/2* mutation. More recently, the PRIMA trial has shown that niraparib is also effective in the overall population regardless of the HRD status, but the post hoc subanalysis has clearly shown that those patients with *BRCA1/2* mutations and other HRDs benefited most from maintenance treatment with niraparib [161].

For patients at high risk for recurrence/progression, there is currently a lack of evidence to suggest the superiority of either anti-angiogenic agents or PARP inhibitors over the other. Interim data from the ongoing PAOLA study investigating the combination of PARP inhibitors and bevacizumab suggest a significant benefit in PFS from concurrent use of both agents [153]. Future studies will need to identify patient groups who benefit most from PARP inhibitors, anti-angiogenic treatment, evolving therapeutics such as immunotherapies or a combination of these, while balancing the benefits and added toxicities from combination treatments.

## Large-scale discovery of therapeutic targets

High-throughput experiments can serve as useful means of discovery in targeted therapy development. These large-scale molecular examinations enable the generation of novel hypotheses regarding putative therapeutic targets to help select candidates for further validation. As HGSC is characterized by a lack of discernible drivers (aside from *TP53*) and extensive heterogeneity, there remains a vast potential for uncovering unanticipated vulnerabilities as novel targeted therapies. High-throughput experiments for therapeutic target identification can be classified in one of two broad themes: molecular profiling to detect aberrantly expressed molecules in tumors or phenotypic screening to determine the molecules important for cancer cell survival.

### Molecular expression profiling

In molecular expression profiling experiments, high-throughput discovery experiments are performed on one or more molecular classes with the underlying assumption that differences in the expression profile of a molecule, or group of molecules (i.e., those associated with a biological pathway), may inform the understanding of disease pathology. As such, the discovery experiments are typically followed by analyses designed to reveal these differences, including differential expression analyses and ontologically informed pathway analyses. Molecular profiling experiments on patient cohorts enable matching clinical data to molecular phenotype which can be especially apt for identification of candidates for targeted therapies. In one such study, Coscia et al. [162] compared the proteomic profiles of platinum-sensitive and -resistant HGSC patient samples revealing cancer/testes antigen 45 (CT45) expression to be predictive of disease-free survival. By establishing a link between demethylating agents and CT45 expression and by linking CT45 to cytotoxic T cell engagement, two potential therapeutic strategies can be devised from these findings.

Molecular expression profiling experiments offers the advantage of not requiring a priori information regarding the pathology of the disease. However, a significant hurdle which lays between molecular expression profiling experiments and novel therapeutics is the potential need to develop novel drug compounds. While there are some measures for druggability which can inform therapeutic target selection, drug development remains a costly process with uncertain success [163]. One strategy to mitigate this challenge is to focus on protein classes for which there is a history of therapeutic intervention. For instance, though kinases are often effective targets for cancer therapeutics, established protein kinase therapies have demonstrated limited utility in HGSC. Recognizing this disconnect, Kurimchak et al. [164] profiled the kinome of primary tumors and PDXs to detect differentially expressed kinases in HSGC revealing a potential therapeutic target, MRCKA.

Surface proteins are another especially useful class of molecules as their accessibility renders them favorable therapeutic targets—evidenced by the fact that over 58% of the known protein targets of FDA-approved drugs are cell surface proteins [165]. However, proteomic workflows which enrich for surface proteins typically require large starting amounts and are therefore not practical or possible for all systems. Despite the challenges associated with surface proteomic workflows, the profiling of the cell surface proteins is well suited for identifying targets for repurposing approved therapeutics and development of new therapeutics. Antibody–drug conjugates (ADC), an emerging class of therapeutics for cancer treatment, enable surface proteins to act as potential therapeutic targets independent of their direct connection to disease pathology [166, 167]. One ADC, IMGN853, which targets folate receptor ɑ, is being evaluated in a phase 3 clinical trial for folate receptor-positive platinum-resistant EOC patients [168]. In this case, although there is some evidence to suggest that targeting folate receptor ɑ alone could have an effect on cancer progression [169], the cytotoxic component of IMGN853 is the maytansinoid compound conjugated to the antibody which targets microtubules.

### Phenotypic screening

Phenotypic screening approaches can be used to identify tumor-specific molecular dependencies as putative targets for therapeutic inhibition. Technological advancements have enabled large-scale molecular perturbations which allow for the functional examination of thousands of molecules in a single experiment. Functional genomic screens entail the use of RNA interference (RNAi) or more recently, clustered regularly interspaced short palindromic repeats (CRISPR)-Cas9 systems coupled with NGS to characterize the genes associated with a phenotype of interest [170, 171]. In a pan-cancer comparison, Cheung et al. [172] performed genome-wide shRNA knockdown screens in 102 cell lines, including 25 ovarian cancer cell lines revealing 54 genes exclusively essential for ovarian cancer viability and proliferation, underlining the utility of functional genomic approaches to identify lineage-specific dependencies. Functional genomic screening can also be used to identify concurrent therapeutic targets that improve chemosensitivity of existing therapies. Fang et al. [173] performed a genome-wide CRISPR knockout screen in an HGSC cell line treated with olaparib to identify targets that mimic HRD. Based on this screen, the authors were able to characterize a gene whose knockout increased the cytotoxic effects and can potentially extend the clinical utility of PARP inhibitors in HGSC.

In contrast to genome-wide interrogations, functional genomics screens can also be conducted on a subset of experimentally relevant genes. To identify novel synthetic lethal targets in *BRCA2* deficient tumors, Mengwasser et al. [174] conducted targeted CRISPR screens in two pairs of isogenic cell lines; one breast cancer pair and one HGSC pair. The isogenic cell lines differed based on the presence of a functional *BRCA2* gene and the screen targeted 380 genes that are involved in DNA damage repair. Interestingly, the authors determined two candidates that not only demonstrated synthetic lethality with *BRCA2* deficiencies but also *BRCA1,* as evident through subsequent investigations. The candidates identified in this study represent potential therapeutic targets for *BRCA*-deficient tumors that are resistant to PARP inhibitors. Likewise, Baratta et al. [175] designed an in vivo shRNA screen to assess the depletion of ~ 800 genes in xenografts of a human HGSC cell line. The screen revealed several candidates essential for proliferation/survival of HGSC. Through further investigations in patient-derived cell lines, one gene was identified as a potential target for *MYCN* overexpressing tumors.

Although functional genomics experiments are advantageous approaches for identifying molecular vulnerabilities for potential inhibition, exploiting these candidates as therapeutic targets can be hindered by the druggability of proteins. The use of high-throughput drug screens is an alternative phenotypic screening approach to identify actionable dependencies. In these experiments, numerous small-molecule compounds, typically with known mechanisms of action, are simultaneously tested against cells to identify novel vulnerabilities. Kenny et al. [176] recently performed a fully robotic screen of ~ 45,000 small molecules in an HGSC organotypic model consisting of one of five HGSC cell lines, primary human stromal cells and extracellular matrix components. Subsequent in vitro and in vivo assays identified three compounds that prevent cancer adhesion, proliferation and invasion suggesting that these compounds can be promising therapeutics for ovarian cancer metastasis. Additionally, drug screens can be used to elucidate indirect mechanisms of targeting undruggable cancer proteins. Zeng et al. [177] screened a small molecule library in two HGSC cell lines to identify alternative methods of down-regulating *MYC*; an oncogene essential when overexpressed in HGSC yet pharmacologically undruggable. This screen revealed a compound that suppressed *MYC* expression through simultaneous inhibition of three specific cyclin-dependent kinases, hence identifying putative targets for *MYC* overexpressing HGSC tumors. A caveat associated with drug screens is that target discovery is restricted to those proteins with existing small molecule inhibitors, thus ignoring the potential of other therapeutic classes such as monoclonal antibodies.

### Integrated target discovery workflows

The use of the aforementioned high-throughput experiments is beneficial as initial steps in therapeutic target discovery. Both approaches enable the concurrent screening of numerous molecules to select potential candidates for further validation experiments. However, as technological advancements improve the capabilities of these platforms, these large-scale experiments can identify hundreds of hits and given the time-consuming nature of molecular biology interrogations, it is often not feasible to individually follow up on every single hit. The use of bioinformatic tools that prioritize targets (e.g., SurfaceGenie [178]) or mining publicly available data, such as TCGA and the Genotype-Tissue Expression (GTEx) project, can help further narrow down candidates, yet well-annotated complete data are not always available for all diseases. Hence, an emerging workflow for therapeutic target discovery is the integration of both high-throughput approaches for a multifaceted characterization of candidates. By leveraging the advantages of these two orthogonal approaches, an integrated workflow can produce a refined list of candidates that are both actionable and essential. Medrano et al. [179] conducted genome-wide shRNA screens and cell-surface characterizations of 27 HGSC cell lines in parallel, resulting in the identification of CD151 as a cell-surface protein that demonstrated essentiality in a subset of HGSC cell lines. Subsequently, the authors performed RNA sequencing to molecularly characterize the discrepancies in response to knockout of the candidate. This study identified both a novel therapeutic target and a molecular marker of target sensitivity, highlighting the utility of integrated high-throughput workflows for HGSC target discovery. Similar target discovery pipelines have proven promising in other cancer settings as well. Martinko et al. [180] used MS to evaluate changes in cell surface expression associated with oncogenic *KRAS* and in parallel, conducted a targeted CRISPR knockdown screen of ~ 1600 membrane proteins to functionally characterize the oncogenic *KRAS* surfaceome. Integration of both datasets resulted in the discovery of CDCP1 as a therapeutic antibody target for *KRAS*-driven cancer cells. Considering the limited success in translating promising targets into beneficial therapies, comprehensive characterization through both expression profiling and functional analyses early in the discovery process may help ensure the selection of robust targets for therapy development.

## Experimental model systems

### Cell lines

Ideal experimental models should accurately reflect tumor biology to ensure maximum translational utility. Given the ease of use and accessibility, immortalized human cancer cell lines are the most widely used models for experimental interrogations of HGSC [181]. However, until recently, the majority of cell lines used in HGSC research were poorly characterized with uncertain histopathological origins. To address these ambiguities, Domcke et al. [182] compared copy-number changes, mutations and mRNA expression profiles of 47 EOC cell lines and 316 HGSC tumor samples examined by TCGA. Strikingly, this extensive evaluation concluded that the most frequently used cell lines in HGSC research poorly recapitulated the genomic and transcriptomic features of HGSC tumors and are likely other EOC histopathologies. The authors recommended an alternative set of cell lines that closely resemble HGSC tumors and thus would be more appropriate as in vitro models. A separate proteomic profiling study of 28 EOC cell lines, two immortalized ovarian surface epithelial cell lines, three primary fallopian tube epithelial cell isolates and eight HGSC tumor tissues revealed distinct groups of cell lines [183]. The majority of cell lines reported to likely represent HGSC as per Domcke et al. [182] clustered with the proteomes of HGSC tumors and fallopian tube samples, further confirming a HGSC histopathology. Additional studies have revealed discrepancies amongst the ability of HGSC cell lines to model tumor metastasis and histopathology in vivo when xenografted [184, 185]. Together, these studies illustrate the disconnect between certain model cell lines and HGSC tumors and highlight the importance of informed cell line selection.

Another caveat of in vitro cell line models is the artificial microenvironment invoked by monolayer growth on plastic and the lack of multicellularity. Three-dimensional (3D) culture has emerged as a step toward bridging the gap between in vitro and in vivo experiments. 3D culture more closely resembles the tumor microenvironment by restoring 3D cell–cell and cell–ECM interactions [186, 187]. Moreover, research groups have successfully demonstrated co-culturing with patient-derived fibroblasts [188] and patient-derived mesothelial cells [189] in HGSC spheroid models to capture the influence of tumor-stromal cross talk on survival and proliferation. Provided that HGSC disseminates through the release of multicellular aggregates into the peritoneal cavity, 3D organotypic in vitro models have been developed to recapitulate significant events in metastasis and gain insights into tumor biology [190]. Nonadherent 3D models have also been used to investigate cancer stem cell populations enriched in disseminated spheroids which are thought to contribute to chemoresistance in HGSC [191]. The use of organ-specific growth factors to model niche environments has enabled the development of ovarian cancer organoid lines that maintain the genomic and histological features of primary tumors and preserve tumor heterogeneity, highlighting their potential utility for precision medicine research [192].

### Genetically engineered mouse models

Genetically engineered mouse models (GEMMs) offer the potential for in vivo tumor investigation. Various molecular biology techniques can be used to introduce genetic modifications in a spatial and temporal manner for in vivo modeling of genetic defects contributing to tumorigenesis [193]. However, the preclinical utility of these models is dependent on the accuracy in recapitulating the histology and pathogenesis of human tumors, an element which has historically proven difficult in the context of HGSC. Given the uncertainty in the site of origin for HGSC, early attempts to develop HGSC GEMMs have focused on targeting the ovarian surface epithelium for genetic manipulations [193]. These models failed to replicate the molecular and clinical features observed in human HGSC tumors. In addition to selecting the correct cell of origin, targeting genes that are relevant to HGSC is also an important consideration when generating GEMMs. Indeed, targeting different combinations of oncogenes and tumor suppressors in the same cell of origin has resulted in different HGSC GEMM phenotypes [194, 195]. Fortunately, as molecular understanding of HGSC biology evolves, so does the ability to correctly model the disease. Targeting HGSC relevant genes, such as *TP53*, *BRCA1*, *RB1* and *PTEN*, in fallopian tube epithelial cells has resulted in a new generation of HGSC GEMMs [195–197]. These clinically relevant models reproducibly demonstrated the formation of precursor STICs in fallopian tubes and mirrored the aggressive metastatic patterns observed in human HGSCs. Hence, if modeled correctly, GEMMs represent promising options for interrogations of early-stage disease and identifying new therapeutic targets.

### Patient-derived xenografts

PDXs are an alternate approach for in vivo experimental models, in which minced fragments of patient tumors are transplanted into immunodeficient mice [198]. The primary advantage of this experimental system is the ability to perform in vivo interrogations of human tumors. Although PDXs have been successfully generated through various different engraftment locations, orthotopic engraftment is preferred as it results in a physiologically relevant microenvironment [199]. In the context of HGSC, there are two engraftment sites that are considered orthotopic: intrabursal (IB) engraftment and intraperitoneal (IP) engraftment [181]. IB engraftment refers to the injection of tumor cells into the ovarian bursa, which is the fat pad surrounding a murine ovary [200]. As there are anatomic differences between the reproductive systems of mice and humans, the bursa can often hinder the extensive peritoneal metastasis characteristic of advanced human ovarian tumors [200]. Alternatively, IP engraftment consists of injecting the tumor cell suspension directly into the peritoneal cavity, mirroring human abdominal tumor dissemination [120, 200]. In addition to recapitulating tumor pathology, orthotopic HGSC PDXs have also been shown to maintain molecular profiles highly comparable to patient tumors [201] and emulate patient-specific responses to platinum-based therapy [202]. These studies highlight the advantage in using PDXs for identifying novel precision medicine approaches in HGSC as it provides an in vivo opportunity for investigating tumor heterogeneity. Indeed, Weroha et al. [203] developed an orthotopic PDX bank consisting of 241 EOC models that reflected the molecular diversity observed in patients and can be a promising resource to investigate subtype specific biomarkers and therapies. A limitation of PDX models is the inability to evaluate interactions between the tumor and the immune system, an important facet of the tumor microenvironment [198].

## Additional considerations for high-throughput studies

Apart from potential unsuitable experimental models, there are several other factors that can impede the utility of high-throughput studies in precision medicine discovery efforts. Provided that several preanalytical variables (e.g., time to freezing, storage duration, serum vs. plasma etc.) have been shown to influence molecular profiles [116, 204], a lack of standardized sample collection and storage protocols can pose as a challenge and potential source of incompatibility for multisite investigations. Though these variables cannot be retrospectively regulated when using samples from biobanks, information about these preanalytical variables should be collected and examined as possible confounders during data analysis. Another consideration is the need for well-annotated clinical cohorts. HGSC patients with extensive disease preventing optimal surgical debulking are often candidates for neoadjuvant chemotherapy [205]; hence, tumor resection is performed only after a round of therapy. As therapeutics can drive the evolution of tumors, tumor samples from these patients likely reflect a different molecular state than those without prior therapy. It is thus essential when conducting high-throughput investigations of clinical samples to use samples with extensive documentation to account for influences from other clinical variables such as treatment history. Additionally, the unprecedented scale of biological data prompts the need for higher computational infrastructure to effectively store and analyze the immense volume of data. While high-throughput experiments are generally well suited for discovery, ultimately to be of clinical benefit, the findings must be integrated into testing regimes which are compatible with the healthcare environment (i.e., cost-effective and quick).

## Conclusions and future perspectives

Through applications in molecular subtyping, liquid biopsies, and targeted therapies, advancements in high-throughput technologies have opened new avenues for precision medicine discovery in HGSC. Large-scale tumor profiling has provided insights regarding the molecular complexity underlying tumorigenesis. Appreciation of this vast heterogeneity has warranted diverging from the one-size-fits-all approach traditionally used for the management of HGSC and EOC as a whole. The use of genetic testing for *BRCA1/2* and other HRDs as an indication for the use of PARP inhibitors is an example of the adoption of precision medicine into the clinical management of HGSC, yet the dismal five-year survival rate suggests that more work is still needed. The ability to simultaneously examine thousands of molecules in a single experiment has fueled the discovery of numerous putative tumor markers for liquid biopsies in HGSC. Considering the limited utility of single markers (e.g., CA125) due to tumor heterogeneity, the use of high-throughput tools has enabled the potential for uncovering multimarker panels with improved clinical performance. Large-scale biological experiments have also been utilized for the identification of novel therapeutic targets, and integration of orthogonal approaches can be promising for the detection of actionable vulnerabilities in HGSC.

Despite the alluring potential of high-throughput approaches, failure to appreciate the intricate nature of HGSC biology in research design and experimental models can stifle the translational utility of the findings from these experiments. Considering that HGSC and the other histological subtypes of EOC are distinct diseases characterized by differences in molecular profiles, clinical progression and pathogenesis, EOC is often still examined as a single entity without subtype-specific stratification in preclinical and clinical validation studies, thus acting as a potential confounder of findings. Furthermore, despite increasing evidence indicating fallopian tissue epithelium as the primary tissue of origin for HGSC, many studies continue to use ovarian surface epithelium as ‘normal’ tissue for comparative experiments, resulting in the potential identification of biologically irrelevant biomarkers and therapeutic targets. As such, it is imperative to incorporate our evolving understanding of HGSC biology in research design to leverage the full potential of emerging high-throughput applications in precision medicine.

## Data Availability

Not applicable.

## Abbreviations

ADC: Antibody–drug conjugate
CA125: Cancer antigen 125
cfDNA: Cell-free DNA
CNA: Copy number alterations
CPTAC: Clinical Proteomic Tumor Analysis Consortium
CRISPR: Clustered regularly interspaced short palindromic repeats
CT: Computed tomography
CTC: Circulating tumor cell
ctDNA: Circulating tumor DNA
EOC: Epithelial ovarian cancer
EV: Extracellular vesicle
FIGO: International Federation of Gynecology and Obstetrics
GEMM: Genetically engineered mouse model
GTEx: Genotype-Tissue Expression
HE4: Human epididymis protein 4
HGSC: High-grade serous carcinoma
HRD: Homologous repair deficiency
IB: Intrabursal
IOTA: International Ovarian Tumor Analysis
IP: Intraperitoneal
LGSC: Low-grade serous carcinoma
miRNA: MicroRNA
MRM: Multiple reaction monitoring
MS: Mass spectrometry
NGS: Next-generation sequencing
OS: Overall survival
PARP: Poly(ADP-ribose) polymerase
PCR: Polymerase chain reaction
PDS: Primary debulking surgery
PDX: Patient-derived xenograft
PFS: Progression-free survival
PRM: Parallel reaction monitoring
RMI: Risk of Malignancy Index
RNAi: RNA interference
ROMA: Risk of Malignancy Algorithm
STIC: Serous tubal intraepithelial carcinoma
TCGA: The Cancer Genome Atlas
VEGF: Vascular endothelial growth factor
